# Trend and impact of international collaboration in clinical medicine papers published in Malaysia

**DOI:** 10.1007/s11192-013-1121-6

**Published:** 2013-09-17

**Authors:** Wah Yun Low, Kwan Hoong Ng, M. A. Kabir, Ai Peng Koh, Janaki Sinnasamy

**Affiliations:** 1 Faculty of Medicine, University of Malaya, Kuala Lumpur, Malaysia; 2 Department of Biomedical Imaging, Faculty of Medicine, University of Malaya, Kuala Lumpur, Malaysia; 3 Department of Applied Statistics, Faculty of Economics, University of Malaya, Kuala Lumpur, Malaysia; 4 University of Malaya Library, Kuala Lumpur, Malaysia

**Keywords:** Bibliometrics, Collaboration, Clinical medicine, Authorship, Citation

## Abstract

Research collaboration is the way forward in order to improve quality and impact of its research findings. International research collaboration has resulted in international co-authorship in scientific communications and publications. This study highlights the collaborating research and authorship trend in clinical medicine in Malaysia from 2001 to 2010. Malaysian-based author affiliation in the Web of Science (Science Citation Index Expanded) and clinical medicine journals (*n* = 999) and articles (*n* = 3951) as of 30th Oct 2011 were downloaded. Types of document analyzed were articles and reviews, and impact factors (IF) in the 2010 Journal Citation Report Science Edition were taken to access the quality of the articles. The number of publications in clinical medicine increased from 4.5 % (*n* = 178) in 2001 to 23.9 % (*n* = 944) in 2010. The top three contributors in the subject categories are Pharmacology and Pharmacy (13.9 %), General and Internal Medicine (13.6 %) and Tropical Medicine (7.3 %). By journal tier system: Tier 1 (18.7 %, *n* = 738), Tier 2 (22.5 %, *n* = 888), Tier 3 (29.6 %, *n* = 1170), Tier 4 (27.2 %, *n* = 1074), and journals without IF (2.1 %, *n* = 81). University of Malaya was the most productive. Local collaborators accounted for 60.3 % and international collaborations 39.7 %. Articles with international collaborations appeared in journals with higher journal IFs than those without international collaboration. They were also cited more significantly than articles without international collaborations. Citations, impact factor and journal tiers were significantly associated with international collaboration in Malaysia’s clinical medicine publications. Malaysia has achieved a significant number of ISI publications in clinical medicine participation in international collaboration.

## Introduction

In last decade, international collaboration has been intensified due to the effects of globalization and rapid development in scientific communication. With international research collaboration, there is an increased in international co-authorship in scientific communications and publications. Internationally co-authored articles have doubled since 1990s and continued to increase until now and in all field of disciplines (Prathap 2013; Wagner 2008; Wagner and Leydesdorff 2005; NSB 2002; Glanzel 2001; Georghious 1998; Dore et al. 1996). It also improves research quality and high impact publications in high impact scientific journals. International co-authored publications have shown to have greater number of citations than domestic or national co-authored publications (Levitt and Thelwall 2009; Glanzel et al. 1999; Katz and Martin 1997). Others have also shown that international collaboration enhances citation impact (Lancho-Barrantes et al. 2013; Moya-Anegon et al. 2008; Chinchilla-Rodriguez et al. 2010; Hsu and Huang 2010). There are cited up to twice as frequently as single-country papers (Narin and Whitlow 1990).

Research in international collaboration and publication is abounding in developed countries. But such is not the case in Asia, where our regional journals are faced with scarce resources like financial issues, peer review system, journal management and operation guidelines and these have to be addressed to compete with more established journal or articles from advanced countries (Low and Ng 2011). One cannot doubt that international collaboration is the way forward to improve quality of the article, its coverage and its impact. Elsewhere in Asia, numerous studies were undertaken highlighting the quality and quantity of scientific publications and international collaboration, particular in China (Wang et al. 2013, He 2009), Taiwan (Liu et al. 2012; Chen et al. 2007) and in India (Basu and Kumar 2000; Kundra and Kretschmer 1999). However, in Malaysia, there is a dire need for international research collaboration and publication as studies in this area is indeed very scanty.

Adams (2013) stressed that we are entering the fourth age of research driven by international collaboration, and institutions that do not form international collaborations risk progressive disenfranchisement, and countries that do not nurture their talent will lose out entirely. Thus, to maintain intellectual strength and to inculcate a good research culture, we need to further study the strengths and weaknesses of research collaboration and publications. Studies such as these are negligible in a country like Malaysia where although there is a lot of emphasis on research collaboration and scholarly publication in achieving internationally recognized university ranking, much has not been known of the output of such collaboration and research funding. Thus, such a study is indeed justifiable in determining the output of research funding and the direction of the country’s publication.

Based on the Malaysian Science and Technology Indicator 2008 report (Ministry of Science, Technology and Innovation Malaysia 2008), the total number of Malaysian-authored Science and Technology (S&T) publications as indexed in SCOPUS from Feb 2001 to Feb 2009 were 22,276. The growth rate of publications was 5 % for 2002 and 13.7 % for 2008. Among the public institutes of higher learning, University of Malaya (UM) produced the highest number of publications in S&T followed by University Science Malaysia (USM), University Putra Malaysia (UPM), University Kebangsaan Malaysia (UKM), and University Technology Malaysia (UTM) (from 2001 to 2008).The total number of publications indexed in Thomson Reuters Web of Science (WoS) database (2001–2010) was 24,377. The contributions according to different broad subject category were: Science (54 %), Engineering (21 %), Medicine (17.5 %), Arts, Humanities and Social Sciences (4 %), Computer Science (3 %), and Dentistry (0.5 %).However, these dataset did not provide a detailed analysis for medicine. Therefore, this paper analyzes Malaysian research collaboration in the field of clinical medicine using some standard bibliometric indicators to examine the pattern and the impact of international collaboration on publication productivity. Specifically, this study aim to examine the trend of international collaboration in scholarly publication in the area of clinical medicine over the years from 2001 till 2010, and also to examine the patterns of such collaboration in terms of journal impact factor, citations impact of both domestic and international articles, collaboration countries and determining the factors influencing international collaboration in scholarly publications.

## Methods

### Data sources

From ISI Web of Science^®^ (including Science Citation Index Expanded, and Social Sciences Citation Index), we downloaded the records belonging to Malaysian based on the addresses of the authors’ affiliations. The time period of our analysis was limited to the publication years between 2001 and 2010. Because the year in the search fields referred to the year in which a paper was indexed, we extended the searched time-spans to 2000–2011, and extracted those eligible records. We also downloaded a journal list that was compiled by the Thomson Reuters Corporation for the ISI Essential Science Indicators^®^. Each of the journals (status: October 2011) in the list was categorized accordingly to the respective fields. The field of clinical medicine of Malaysian based authors contained 990 journals; these journals were used to identify bibliographic records belonging to clinical medicine and found 3941 articles.

### Study design

The analysis was limited to the “articles” and “reviews” in the journal of clinical medicine covered in the ISI database. Notes, letters, editorials, news, meeting abstracts were excluded from the analysis. The impact factor (IF) in the 2010 Journal Citation Report (JCR) Science Edition were taken to access the quality of the articles. We identified the authors’ affiliations and countries from the fields of affiliation and corresponding address. International collaboration was deemed to exist in an article if any author’s affiliation was located outside Malaysia. The names of affiliations were less well formatted than those of countries. Besides, an institution might change its name during the study period or have several affiliates. This required the authors to process the affiliations manually.

We computed the publication counts and the share of articles with international collaboration in each year. Moreover, the data were then stratified according to journal impact factor, subject category, domestic institution and collaborating country in periods (2001–2005, 2006–2010) and also for whole set 2001–2010. The subject category that was generally used in the compilation of the annual Journal Citation Reports^®^ was presented in each ISI record. One journal with its articles might be indexed with several subject categories. To accredit an article to institutions and countries, we adopted the method of “absolute country counting”, in which each institution or country contributing to an article received one paper credit, respectively.

### Data processing and statistical analysis

#### Univariate and bivariate analysis

The extraction and computation of data was undertaken with the Perl programming language (version 5.8.7, http://www.perl.com/). We computed the descriptive statistics, e.g. the frequency in count and their percentages. Moreover, we computed the associations between Malaysian’s number of articles and the share of articles with international collaboration in each year, and the journal impact factor with 7 groups (<1, ≥1&<2, ≥2&<3, ≥3&<4, ≥4&<5, ≥5 and journal without impact factor). Furthermore, we also computed the association between Malaysia’s clinical medicine publications in the WoS and the share of articles with international collaboration by subject category (selected), Malaysian’s top public institutions (selected), and selected top international collaborating countries in periods (2001–2005, 2006–2010) and for whole study period (2001–2010).

### Multivariate analysis: classification and regression trees (CART)

**Fig. 1:**
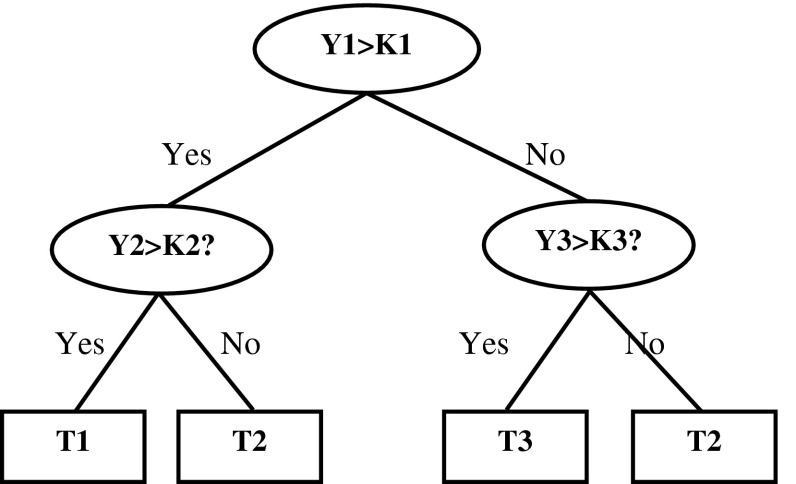
A typical CART model for classification. *Oval* nodes are the intermediate nodes and *rectangles* are terminal nodes. K1, K2, and K3 are splitting values of the variables Y1, Y2, and Y3

CART is a data exploration and prediction algorithm. To develop a CART, each predictor is chosen based on how well it fits separately the records with different predictions. The entropy metric (Witten and Frank 2005) is used to determine whether a split point for a given predictor is better than the other. The CART algorithm has divided the independent variable into two separate hyper-rectangular areas according to performance measures (Dunham 2003; Hastie et al. 2001). In algorithmic point of view, CART has a forward stepwise technique that adds model terms and a backward technique for pruning, and selects important variables that are useful in the model. The output of the model is hierarchical structure that consists of a series of if–then rules to predict the outcome of the dependent variable (Moon et al. 2012). For example, at each intermediate node (ovals in Fig. 1) of the decision tree, a question is asked about the variables (e.g., Y1, Y2, and Y3) of data.

The data set that satisfies the question (inside ovals) goes left in the branching and right if it fails to meet the criterion. Based on splitting values (e.g., K1, K2, and K3) of the variables, every data point ends up in one of the nodes called terminal nodes (Ti’s) (rectangles in Fig. 1) of the tree. It can then determine the criteria for each terminal node by retreating up the tree to the top node. For instance, in Fig. 1, the first terminal node (terminal node farthest to the left) retreats up the left edge of the tree, yielding the following rule: ‘‘If Y1 > K1 and Y2 > K2, then it will be classified as T1 (first terminal node).’’ Other terminal nodes in the tree can be interpreted similarly. All the statistical analyses were performed using SPSS for Windows Release 20.0 (SPSS Inc., Chicago, IL, USA).

## Results

### Univariate and bivariate

**Fig. 2:**
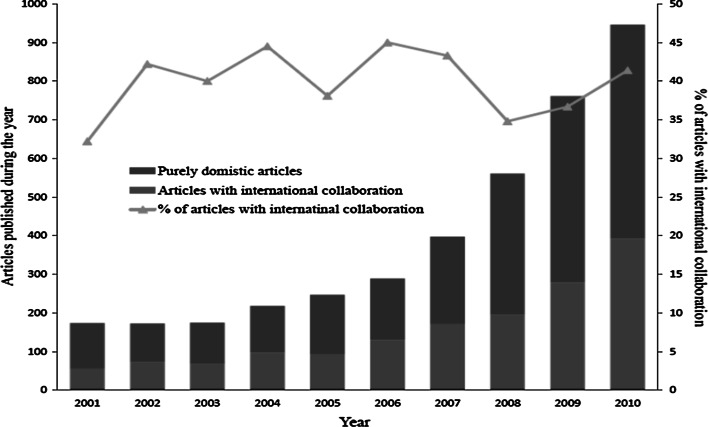
Trends in Malaysia’s clinical medicine publications in the WoS

A total of 3,941 published articles on clinical medicine were retrieved from the WoS between 2001 and 2010 where at least one author was from Malaysian institutions. Malaysia’s ISI publications in clinical medicine increased from 174 articles in 2001 to 946 in 2010, and the articles with international collaboration from 56 in 2001 to 392 in 2010 (Fig. 2). The percentage of articles with international collaboration varied between 32.2 and 45 % annually.

Among 3,941 articles in 990 journals, 81 articles were not linked with JCR. Based on JCR, only 213 (5.4 %) articles were published in high impact journals (journals with an impact factor ≥5) whereas, 2,269 (about 58 %) articles published in journals impact factors less than 2 (Table 1). The articles with international collaboration and the journal impact factors were found statistically significant at *p* < 0.001 and the probability of international collaboration increased with journal impact factors. For instance, more than 78 % articles with high impact factor (IF ≥5) were from international collaboration in contrast with 22 % articles with journal impact factor less than 1. On the average, articles published with international collaboration appeared in journals with high impact factor than those without collaboration.

**Table 1:** Distribution of Malaysia’s clinical medicine publications in the WoS by journal impact factor and the international collaboration, 2001–2010

Journal impact factor^a^	No. of published articles	Articles with international collaboration, *n* (%)	Chi square (d.f) (*p* values)
<1	1,168	252 (21.6)	489.26 (6) *p* < 0.001
≥1&<2	1,101	370 (33.6)	
≥2&<3	909	454 (49.9)	
≥3&<4	308	188 (61.0)	
≥4&<5	161	113 (70.2)	
≥5	213	167 (78.4)	
NA	81	14 (17.3)	
Total	3,941	1558 (39.5)	

*NA* not applicable

^a^The journal impact factor was based on the 2010 Journal Citation Reports Science Edition

The Malaysia’s clinical medicine publications were indexed in 41 subject categories. Table 2 presents 32 subject categories with high frequency of articles and another category combining (allergy, critical care medicine, dermatology, emergency medicine, geriatrics and gerontology, nursing, rehabilitation, substance abuse, and transplantation)was created as ‘other category’. These subject categories were presented in 5-year intervals (2001–2005 and 2006–2010) as well as 2001–2010. The number of articles in each of 33 subject categories did not correlate with % of articles with international collaboration Kendall’s tau-b (=0.019, *p* = 0.159 for 2001–2010; −0.009, *p* = 0.725 for 2001–2005 and 0.029, *p* = 0.067 for 2006–2010).

**Table 2:** Distribution of Malaysia’s clinical medicine publications in the WoS by subject category (selected)

Subject category	2001–2010		2001–2005		2006–2010	
	Total no.	Articles with IC, *n* (%)	Total no.	Articles with IC, *n* (%)	Total no.	Articles with IC, *n* (%)
Anesthesiology	22	2 (9.1)	15	2 (13.3)	7	0 (0)
Cardiac and cardiovascular systems	29	22 (75.9)	8	5 (62.5)	21	17 (81.0)
Clinical neurology	153	71 (46.4)	40	17 (42.5)	113	54 (47.8)
Endocrinology and metabolism	90	51 (56.7)	23	11 (47.8)	67	40 (59.7)
Gastroenterology and hepatology	108	49 (45.4)	38	11 (28.9)	70	38 (54.3)
Hematology	37	13 (35.1)	12	3 (25.0)	25	10 (40.0)
Imaging science and photographic technology	33	19 (57.6)	7	3 (42.9)	26	16 (61.5)
Immunology	124	54 (43.5)	48	19 (39.6)	76	35 (46.1)
Infectious diseases	146	81 (55.5)	48	22 (45.8)	98	59 (60.2)
Integrative and complementary medicine	63	11 (17.5)	15	2 (13.3)	48	9 (18.8)
Medicine, general and internal	536	138 (25.7)	46	18 (39.1)	490	120 (24.5)
Medicine, research and experimental	138	64 (46.4)	19	11 (57.9)	119	53 (44.5)
Neurosciences	43	30 (69.8)	10	8 (80.0)	33	22 (66.7)
Obstetrics and gynecology	117	26 (22.2)	28	2 (7.1)	89	24 (27.0)
Oncology	230	102 (44.3)	32	18 (56.2)	198	84 (42.4)
Opthalmology	67	34 (50.7)	14	7 (50.0)	53	27 (50.9)
Orthopedics	22	16 (72.7)	5	2 (40.0)	17	14 (82.4)
Otorhinolaryngology	56	8 (14.3)	13	4 (30.8)	43	4 (9.3)
Pathology	118	49 (41.5)	48	17 (35.4)	70	32 (45.7)
Pediatrics	140	41 (29.3)	62	18 (29.0)	78	23 (29.5)
Peripheral vascular disease	22	13 (59.1)	9	6 (66.7)	13	7 (53.8)
Pharmacology and pharmacy	549	237 (43.2)	143	60 (42.0)	406	177 (43.6)
Physiology	39	31 (79.5)	14	11 (78.6)	25	20 (80.0)
Psychology, clinical	21	15 (71.4)	3	1 (33.3)	18	14 (77.8)
Radiology, nuclear medicine and medical imaging	70	31 (44.3)	23	8 (34.8)	47	23 (48.9)
Respiratory system	21	7 (33.3)	13	3 (23.1)	8	4 (50.0)
Rheumatology	40	15 (37.5)	4	3 (75.0)	36	12 (33.3)
Surgery	165	51 (30.9)	54	15 (27.8)	111	36 (32.4)
Toxicology	150	61 (40.7)	51	20 (39.2)	99	41 (41.4)
Tropical medicine	289	78 (27.0)	33	16 (48.5)	256	62 (24.2)
Urology and nephrology	92	42 (45.7)	38	13 (34.2)	54	29 (53.7)
Virology	113	51 (45.1)	48	21 (43.8)	65	30 (46.2)
Others^a^	98	45 (45.9)	23	13 (56.5)	75	32 (42.7)
Total	3941	1558 (39.5)	987	390 (39.5)	2954	1168 (39.5)

Chi square statistics, d.f, *p* values, Kendall’s tau-b coefficient

*IC* international collaboration

^a^Articles with <0.5 % in subject categories are merged in other category

Among the top five public institutions publishing articles in the ISI journals, UM was the most productive (1,238 articles; 31.4 %) during 2001–2010 followed by USM (851 articles; 21.6 %) and UKM (655 articles; 16.6 %). However, the publications increased among the top institutions from 2001–2005 to 2006–2010 were UM (2.4 times), USM (3.6 times), UKM (2.9 times), UPM (3.2 times) and IMR (4 times).

Overall (2001–2010), articles published with international collaboration was high among UM researchers (41.2 %) than other public institutions. However, in 2001–2005, three institutions namely UM, USM and IMR have the same percentage (more or less 37 %) of published articles with international collaboration though publications varied in numbers with the highest publication from UM researchers (Table 3). In 2006–2010, UM also has the highest role in terms of % share of clinical medicine publications as well as the number of publications in the ISI WoS.

**Table 3:** Distribution of Malaysia’s clinical medicine publications in the WoS by institution (selected)

Institution	2001–2010		2001–2005		2006–2010	
	Total no.	Articles with IC, *n* (%)	Total no.	Articles with IC, *n* (%)	Total no.	Articles with IC, *n* (%)
University of Malaya (UM)	1238	510 (41.2)	359	136 (37.9)	879	374 (42.5)
University of Science Malaysia (USM)	851	267 (31.4)	185	68 (36.8)	666	199 (29.9)
University Kebangsaan Malaysia (UKM)	655	175 (26.7)	167	49 (29.3)	488	126 (25.8)
University Putra Malaysia (UPM)	441	138 (31.3)	105	33 (31.4)	336	105 (31.2)
Institute of Medical Research (IMR)	178	50 (28.1)	35	13 (37.1)	143	37 (25.9)

Chi square statistics, d.f, *p* values, Kandall’s tau-b coefficient

*IC* international collaboration

Based on co-authorship of articles, the Malaysian clinical medicine researchers has collaborated with countries and regions but only 11 countries have published more than 90 articles. The percentage distribution of other collaborating countries showed a scattered pattern. About 52 % articles were published with 11 major collaborating countries. The Malaysian publications with major collaborating countries have increased from 556 articles (27 %) in 2001–2005 to 1,487 articles (73 %) in 2006–2010 (Table 4).

**Table 4:** Distribution of Malaysia’s clinical medicine publications with international collaboration in the Web of Science by collaborating country (selected)

Collaborating country	2001–2010	2001–2005	2006–2010
United States of America	297 (7.5)	90 (9.1)	207 (7.0)
Great Britain	286 (7.3)	76 (7.7)	210 (7.1)
Australia	269 (6.8)	64 (6.5)	205 (6.9)
Singapore	232 (5.9)	61 (6.2)	171 (5.8)
Japan	223 (5.7)	73 (7.4)	150 (5.1)
China	178 (4.5)	50 (5.1)	128 (4.3)
India	159 (4.0)	29 (2.9)	130 (4.4)
Thailand	128 (3.2)	29 (2.9)	99 (3.4)
Indonesia	91 (2.3)	29 (2.9)	62 (2.1)
Korea	90 (2.3)	25 (2.5)	65 (2.2)
Taiwan	90 (2.3)	30 (3.0)	60 (2.0)
Total	2043 (100.0)	556 (27.2)	1487 (72.8)

Percentages are in parenthesis

Among the collaborating countries, USA and Great Britain were the top collaborators with contribution more than 7 % of the published articles. Between two periods, these two countries have dramatically increased their collaborations with Malaysian researchers. Moreover, the significant collaboration was found from Australia (6.8 %), Singapore (5.9 %) and Japan (5.7 %). In addition, the other collaborating countries also have sizable publications with Malaysian researchers (Table 4).

### Multivariate results

Classification and regression tree (CART), a data mining technique was used to estimate the collaboration status, i.e. the response variable. Model specification showed that ten-fold cross validation was used to predict the true misclassification rate of independent variables namely, citations, journal impact factors, and journal tiers. Moreover, the maximum tree depth, minimum cases in the parent and child nodes were 5, 250, and 150 respectively. Model results showed that total number of nodes, number of terminal nodes and tree depth were 9, 5, and 3 respectively. The degree of association between collaboration status and independent variables were examined using Chi square test. The independent variables, citations (χ^2^ = 201.0, *p* < 0.001), impact factor (χ^2^ = 372.35, *p* < 0.001), and journal tiers (χ^2^ = 324.21, *p* < 0.001) were found to be significantly associated with international collaboration of Malaysia’s clinical and medicine publications. The classification accuracy of each class of the response variable showed satisfactory results. Moreover, the overall classification accuracy was about 70 %, demonstrating that the constructed decision tree model performs reasonably well for predicting the response variable, i.e. the collaboration status. For variable selection, CART software provides “variable importance scores.” The variables that receive a 100 score indicates the most influential independent variable for predicting the dependent variable, followed by other variables based on their relative importance to the most important one. The journal tiers and journal impact factors showed the most important independent variables with normalized importance 100 and 98 respectively. However, citations showed the normalized importance of slightly over 65.

**Fig. 3:**
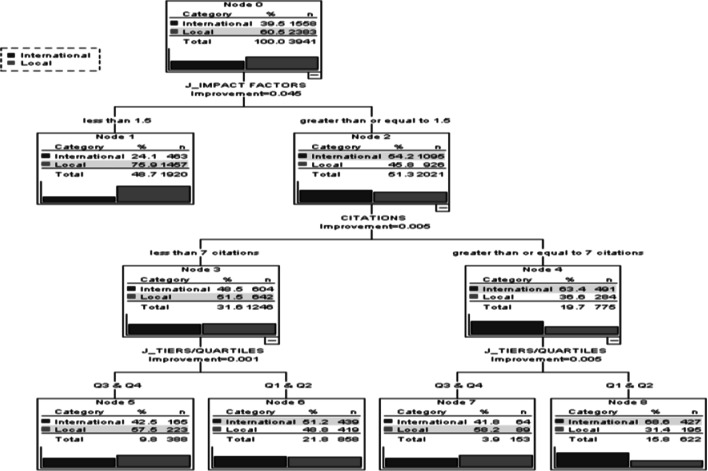
The CART model for predicting the collaboration status of Malaysia’s clinical medicine publications in the ISI WoS

The CART algorithm builds a tree model (Fig. 3) by splitting the independent variable space into regions. The final regions are called the terminal nodes (node 1 in this tree model is the first terminal node). Each node of the tree specifies conditions that split an existing region. For instance, from the first terminal node, “if articles published in journals impact factor less than 1.5, only 24 % were from international collaboration.” In general articles with high impact factors comes from international collaboration. However, it is interesting to discover from our tree result that articles published in high impact (>1.5) journals 54.2 % comes from international collaboration. Decision tree results also indicated that articles with <7 citations 48.5 % comes from international collaboration whereas articles with ≥7 citations, 63.4 % comes from international collaboration. This way we can discuss all the terminal nodes. For example, in node 8 (last terminal node), we can say, “if articles published in journals IF ≥1.5, citations ≥7 and journals were from first and second tiers/quarters, 69 % were from international collaboration compared to only 31 % articles were from purely domestic address.”

## Discussion

This is the first study focusing on collaboration in clinical medicine in Malaysia, which is also the first of such kind of study in Asia. In the Asian region, articles on the analysis of collaboration pattern mainly in non-clinical medicine have been published by China (Wang et al. 2013; Wang et al. 2005; Guo et al. 2000), India (Basu and Aggarwal 2001; Kundra and Kretschmer 1999)and Taiwan (Chen et al. 2007; Liu et al. 2012), but none in Malaysia.

Our study found that the number of articles and reviews in Malaysia’s clinical medicine publications has increased during the study period (2001–2010). The majority of the published papers were in Tier 3 (Q3) and Tier 4 (Q4) and of IF <1. By subject category, the majority of papers were from the categories of Pharmacology and Pharmacy, Medicine-Internal and General, and Tropical Medicine. In Malaysia, the top five institutions publishing papers in clinical medicine journals are: UM, USM, UKM, UPM and IMR. Over the years, we have also seen the number of articles increased. The UM being the premier university in the country still remains the top in research productivity and publications. In India, Indian authors’ participation in co-authored publications in the Indian Medical Sciences showed an increasing trend (Kundra and Kretschmer 1999).

The proportion of internationally collaborated articles in Malaysia’s clinical medicine publications remains low during the study period. It was evident that the number of articles published in each year correlated significantly with the percentage of articles with international collaboration (*p* < 0.001). International collaboration papers increased with journal Tiers 29.5 % in articles with Tier 1 journals in contrast with 16.2 % in articles with Tier 4 journals. Over the years, we are seeing articles produced in high-impact journals. This could be due to the government support in research funding and incentives given by the University in promoting international scientific collaboration in research and publication in top quality journals. Others have also shown an inflation in internationally co-authored articles (Leydesdorff and Wagner 2008; Persson et al. 2004). Similarly in Asia, there has been an increase trend in international scientific collaboration in publication. For example, the number of Chinese publications had exponential growth, at the same time; international collaboration publication also had exponential growth (Jin and Rousseau 2007).

Articles with international collaborations appeared in journals with high impacts than those without international collaboration (mean 3.04 vs. 1.52 respectively, *p* < 0.001). Basu and Aggarwal (2001) argued that foreign collaboration not only increases the overall impact of an institution but also significantly changes the overall impact of the institutional output. They further proposed an index of gain in impact thorough foreign collaboration. Prathap (2013) put forth an index of foreign collaboration, where it measures the extent to which co-publication through international collaboration enhances the value of scientific output of an organization. These internationally co-authored papers had significantly higher impact factors. Articles with international collaborations were cited more frequently than articles without international collaborations (mean 10.81 vs. 4.08 respectively, *p* < 0.001). Similarly others have also found that internationally co-authored articles appear to be cited more often than nationally co-authored papers (Lancho-Barrantes et al. 2013; Chinchilla-Rodriguez et al. 2010; Moya-Anegon et al. 2008; Leimu and Koricheva 2005; Persson et al. 2004; Narin 1991).

Among the collaborating countries in our study, USA and Great Britain, the most important collaborating partners of Malaysia’s clinical medicine research contributed to 7.7 % of published articles. Collaboration with the United States and Great Britain played an important role in Malaysian research collaboration. Similarly in China (Wang et al. 2013), although not specifically in the field of clinical medicine, nearly 95 % international co-authored Chinese papers are collaborated with only 20 countries and of which almost half are with the United States (Wang et al. 2013; He 2009). USA is the most important collaboration country and the international collaboration between China and the G7 countries display differences at each research field and China has emerged as the regional leader in collaboration (He 2009). Chinese immigrants played an important role in China’s scientific collaboration, especially in English-speaking countries (Wang et al. 2013). Worldwide, the exponential growth of the number of addresses of internationally collaborating authors suggests that the grown of the network extends to many more places around the globe and the average number of addresses in any one internationally co-authored publication has grown from an average of 2.86 in 1990 to 3.61 in 2005, and this trend is accelerating (Leydesdorff and Wagner 2008). As there is globalization in research, there is a flow of knowledge and information between scientists and researchers across the globe, and thus one would see more research collaborations and international co-authored publications. Further, there is a rapid demand for international cooperation in publishing in the Universities as one strives for research excellence.

## Conclusion

The analysis has shown that international collaboration was a common practice in clinical medicine research and that it has contributed to the publication of papers in high impact journals. Our analysis has shown that a substantial percentage of successful publication in high impact journals depended on international collaboration. There is a need to understand the dynamics of international collaborative research so as to formulate best practices for collaborative work in journal publication. One cannot discount the importance of international research and development network between scientists all over the globe. The findings of this study will inform leaders of higher learning institutions and policy makers of its financial resource, research facilities and manpower allocation based on research outputs. More research is warranted on international collaboration in journal publishing in the Malaysian situation in order for us to compete with world class research universities and thus more research funding and investment is needed to support this endeavor. As noted by Adams (2013) in his recent publication in Nature on the Fourth Age of Research, that in emerging economy, the strength of publication is in its domestic output, whereas for established economies, the national research output is due to international collaboration. Thus in order to compete with these established economies; we need to further enhance our talents in the international arena and to build better, resilient and sustainable scholarly communities in higher learning and research institutions.
